# Video Conference Technology as a Tool for Pair Introduction in Rhesus Macaques

**DOI:** 10.3390/ani12141783

**Published:** 2022-07-12

**Authors:** Cara Stull, Allison Heagerty, Kristine Coleman

**Affiliations:** Animal Resources & Research Support, Oregon National Primate Research Center, Oregon Health & Science University, Beaverton, OR 97006, USA; candell@ohsu.edu (C.S.); heagerta@ohsu.edu (A.H.)

**Keywords:** socialization, pair housing, video conferencing, welfare, rhesus macaque, *Macaca mulatta*

## Abstract

**Simple Summary:**

While pair housing macaques is known to improve welfare over single housing, the process by which animals are socialized can be stressful if the animals do not get along. To assess whether or not potential partners are compatible, they typically need to be located in visual contact with one another, which often involves moving one or both animals to a new location. Such relocations can cause stress for animals, particularly if the introduction attempt is not successful. In this study, we examined whether allowing rhesus macaques to interact via video conferencing technology (Zoom) could help us determine compatibility before they were relocated. We provided a Zoom session between partners for 42 pairs of monkeys and coded their behavior. We then examined whether these behaviors predicted future pair success. The monkeys in our study spent surprisingly little time attending to the screen, and attention did not predict pair success. However, the similarity of attention shown by individuals in the pair (e.g., amount displayed by the partners relative to one another) did. Pairs in which attention was primarily shown by one animal were more likely than others to be successful. While additional work is needed, video conferencing technology may help staff determine compatibility between potential partners.

**Abstract:**

Pair housing is known to promote welfare for macaques in captivity. However, finding compatible partners can be challenging, particularly when animals are not located near one another. Because macaques show interest in videos of conspecifics, we examined the use of video conference technology (Zoom) as a potential tool to assess compatibility in 84 rhesus macaques (2–22 years old) prior to pair introduction. Monkeys involved in the pairs (12 female–female, 21 male–male, 9 female–male) were unfamiliar with each other. We set up a 10 min Zoom session between potential partners (on an iPad in front of the cage). We scored attention to the screen, anxiety, and prosocial behaviors and examined whether these behaviors predicted future pair success. Monkeys spent relatively little time attending to the tablet (median = 13.3%), and attention did not predict pair success (B = −0.06, NS). However, pairs in which attention was primarily shown by one animal had a higher chance of success than those in which both individuals showed similar levels (B = −4.66. *p* = 0.03). Neither prosocial (B = 0.89, NS) nor anxiety (B = −1.95, *p* = 0.07) behavior correlated with pair success. While preliminary, our data suggest that video conferencing technology may be useful as a tool for introducing unfamiliar partners prior to a socialization attempt.

## 1. Introduction

Social housing is an essential tool for promoting the psychological wellbeing of nonhuman primates (NHPs) in research. Studies have shown a variety of physiological and behavioral benefits associated with socialization (e.g., [1,2,3,4,5]). Social housing, including continuous full-contact pair housing, provides opportunities for macaques and other NHPs to engage in species-typical behaviors such as grooming and social play [3] and provides a social buffer against environmental stressors [6]. Further, full-contact pair-housed macaques have also been shown to exhibit fewer abnormal behaviors when compared to their single-housed counterparts [7,8].

The process of pair introduction is often performed in a series of steps [8], beginning with partner selection. Several studies have identified attributes associated with compatibility between macaque partners, including weight [8,9,10,11,12], age [11,12], temperament [9,13], and previous experience [8]. Once potential partners are determined, they are typically housed next to one another for a period of time, during which they are given various degrees of visual and/or physical contact. For example, partners may be separated with either a mesh or clear plexiglass partition, allowing visual but no physical contact, or with a “protected contact” partition that allows limited tactile contact. At some point, based on the behavior of the monkeys, the partition is removed, allowing full physical contact between the partners.

The decision about whether to proceed with the social introduction is usually based on the observed interactions between the partners; introductions in which the two partners display many affiliative behaviors toward one another are likely to proceed, while those in which the partners engage in aggressive or overly fearful behavior may be aborted. In order to interact, the monkeys clearly need to be in visual contact with one another. However, in many primate facilities, potential partners may be housed in separate rooms or even different buildings from one another, necessitating the relocation of one or both partners for the introduction. Being moved to a new and unfamiliar environment can be stressful for macaques [14,15], and this stress can be compounded if the pair introduction is unsuccessful. In some situations, social introduction attempts may even require the relocation of other monkeys, in addition to those intended for pairing. Therefore, having the opportunity to assess initial behavior *before* moving the monkeys may decrease the need for relocations and reduce the stress associated with social introductions.

Video conferencing technology offers the opportunity for animals to communicate virtually. Many organizations have dramatically increased the use of video conferencing in the past couple of years to improve communication, and there is evidence to suggest that macaques may also be able to communicate in this way. Studies have demonstrated the ability of rhesus macaques to recognize and process faces, particularly facial expressions, of conspecifics in still images by measuring activation in the amygdala [16,17]. Macaques have also been shown to respond to videos of conspecifics. Rhesus macaques watching unedited videos of unfamiliar conspecifics elicited social engagement with the monkey on video, including looking patterns (gaze aversion and gaze following) and reciprocated facial expressions [18]. The animals in that study were not provided with treats or other rewards for looking at the screen, suggesting that they were interested in the video and that their reactions were spontaneous [18]. Further, studies have found that both pre-recorded and live videos of unfamiliar conspecifics were effective rewards for bonnet macaques performing a joystick task [19,20]. Taken together, these studies suggest that macaques can recognize and respond to the behavior of conspecifics on video. While promising, the monkeys in those studies were not able to interact with conspecifics in real time. Technologies such as video conferencing provide opportunities for animals to engage in real-time interactions with a conspecific on a video screen.

In this study, we examined the use of the Zoom video conference app (on tablets) as a potential tool to assess pair compatibility in male and female rhesus macaques prior to relocation for pair introduction. Given that prosocial behavior exhibited early in the introduction can predict pair success [21], we hypothesized that pairs in which the partners demonstrated affiliative behaviors during the Zoom session would be more likely to be successfully pair-housed than those in which there was no prosocial behavior exhibited.

## 2. Materials and Methods

### 2.1. Subjects

Subjects for this study were 84 Indian-origin rhesus macaques (*Macaca mulatta;* 51 males, 33 females), 2.3–22.1 years old (mean age = 8.8 +/− SD 5.14 years), housed at the Oregon National Primate Research Center (ONPRC). Each monkey had been identified as a potential partner in a socialization attempt by the ONPRC behavioral management team based on factors such as age, weight, and temperament. Potential partners were provided with a Zoom conference session prior to the pair attempt (see below). Pairs were either isosexual (12 female–female, 21 male–male) or heterosexual (*n* = 9), but in all cases, the partners were unfamiliar with one another and were housed in different rooms. At the time of the Zoom session, monkeys were single-housed (i.e., housed without physical contact with another individual) in standard, appropriately sized primate cages based on their weight, in accordance with the *Guide for the Care and Use of Laboratory Animals* [22]. Individual cage sizes varied but generally ranged from 24.2–32 inches wide × 27.1–33.5 inches deep × 32.25–36 inches tall. Monkeys were kept on a 12:12 h light cycle and provided monkey chow (Purina LabDiet) twice daily with supplemental daily produce. Water was provided ad libitum through an automatic watering system. All monkeys participated in the ONPRC Behavioral Management Plan. This study was approved by the ONPRC Institutional Animal Care and Use Committee. The ONPRC is fully accredited by AAALAC International and compliant with the United States Animal Welfare Act Regulations [23].

### 2.2. Zoom Video Conferencing

To provide potential partners with visual access to one another prior to the pair introduction, we set up a video conference session using Zoom. For each Zoom session, an observer who was unfamiliar with the monkeys set up an iPad (Mini, Apple, Inc., Cupertino, CA, USA) approximately 24 to 36 inches from the front of the cage. The iPad was secured on a tripod that was then positioned so that the screen was at eye level of the viewing monkey and at a distance that allowed the tablet’s camera to capture a full view of the cage front. While tablets are used for enrichment at the ONPRC, most monkeys were unfamiliar with the iPads. Zoom sessions were 10 min in duration, similar to other studies examining the behavior of pair-housed monkeys [21,24], and were captured using the screen record function on the iPads. They were conducted at various times throughout the day, depending on other activities (husbandry and/or research-related) occurring in the animal rooms. A trained observer scored these sessions using instantaneous and all occurrence sampling [25] every 20 s during the 10 min period. To determine whether the monkeys were attending to the iPad, we scored attention to screen (“attention”) with instantaneous sampling. We also recorded anxiety (i.e., yawning, scratching, and body shake [26,27]) and prosocial behaviors (lip smacking and presenting). Because anxiety and prosocial behaviors were relatively rare, they were scored using all occurrence sampling (i.e., we recorded each time the behavior occurred during the 10 min period). Aggression toward the screen (threats) was rarely observed and therefore not included in the analysis.

### 2.3. Social Introductions and Observations

Social introductions began within 41 days of the Zoom session (“lag time”; mean length of time = 6.48 +/− SD 10.7 days). We attempted to keep this lag time under 2 weeks, but it was not always possible due to schedule changes and unexpected delays. For the introduction, one or both animals were relocated so that the monkeys were housed in adjacent cages and provided with visual contact through a mesh panel. Monkeys were carefully monitored for fear or aggression by trained observers. If there were no overt signs of incompatibility during this phase (e.g., fighting through mesh panel, extreme fear/avoidance, frequent anxious displays), staff proceeded with the introduction, either by removing the mesh panel altogether or replacing it with a protected contact slide (a panel with vertical bars about 2 inches apart, which allows for some physical contact). Time with protected contact lasted 24 h to several days, occasionally longer. All pairs in this study progressed from protected contact to a full-contact introduction.

### 2.4. Statistical Analyses

#### 2.4.1. Relationship between Individual Demographic Data and Behavior on Zoom

Data were analyzed on both the individual and pair level. We first examined whether demographic variables (e.g., sex, age) predicted the display of certain behaviors during the Zoom session. Sex was characterized as sex of the subject to sex to the recipient; i.e., female to female (FtoF), male to male (MtoM), male to female (MtoF), and female to male (FtoM). Age was included as a numerical value in years, measured to the tenth decimal place. We calculated the amount of time each individual showed attention to the screen and the number of times an animal displayed anxiety behavior and prosocial behavior during the 10 min Zoom session.

To determine whether demographics predicted certain behavioral responses to the Zoom sessions, we ran generalized linear mixed models with a Poisson distribution for each behavior (attention, anxiety, and prosocial behavior), testing age and sex as predictor variables (R version 4.0.3, package “lme4”). To account for multiple comparisons (i.e., 3 behaviors), we adjusted our alpha using the Bonferroni correction. The adjusted alpha was 0.017. The unique Zoom pair was included as a random effect to account for repeated sampling within the same pair. Models were compared using Akaike’s information criteria (AIC) scores. A model was considered substantially better than another model if its AIC score was at least 2 points less than the other model [28]. Models were also compared using chi-square tests to further confirm the model choice. Tukey’s honestly significant difference (HSD) contrasts were used to determine differences between sex categories if not apparent in the final model (R version 4.0.3, package “multcomp”).

#### 2.4.2. Relationship between Behavior on Zoom and Pair Outcome

The primary focus of this analysis was to examine whether behaviors displayed by the pair during the Zoom meeting could predict future pair success. To examine this question, we characterized attention, anxiety, and prosocial behavior in two ways: (1) the sum of behavior shown by the pair and (2) the similarity of each behavior between individuals of the pair. The quantification of behaviors within the pair as similar/dissimilar was an a posteriori decision of how to analyze the behaviors in relation to pair success and was based on prior studies indicating that individuals with similar temperaments are more likely to be socially compatible [13]. Thus, we expected that individuals with similar levels of anxiety, prosocial behavior, and attentiveness would be more likely to be successfully paired than those with differing levels of these behaviors. To categorize within-pair similarity for each behavior, the sum of the behavior for an individual was divided by the total for the pair. The absolute value of the difference between that proportion was then categorized as being dissimilar (behavior was disproportionately shown by one animal) if they were greater than or equal to the median value and similar (behavior was shown approximately equally/evenly by both animals) if they were less than the median value. Additionally, a variable capturing the number of days between the Zoom session and when the partners first had live visual contact (i.e., “lag time”) was included in all models.

Pair success was defined as introductions in which the animals remained compatibly co-housed for at least 28 days. To determine whether behaviors (attention, anxiety, and social behavior) exhibited by the pair during the Zoom sessions predicted success, we used logistic regression with the pair as the unit of analysis and an alpha value of 0.05. A model of Zoom behavior on pair outcome was built in 3 steps. First, we began by modeling the effect of each behavior (attention, anxiety, prosocial behavior) on pair outcome separately, using their sum and within-pair similarity variables, with the sex of the pair, the mean age of the partners, the age difference between individuals of the pair, the lag time between Zoom session and pair introduction, and interactions of sex and age with the behavior variables. The variables from the best of each single-behavior model were then combined into an initial comprehensive model. Lastly, nonsignificant variables were removed from this comprehensive model until we reached a minimum AIC score.

## 3. Results

### 3.1. Relationship between Demographic Variables and Behavior on Zoom

#### 3.1.1. Attention

**Figure 1:**
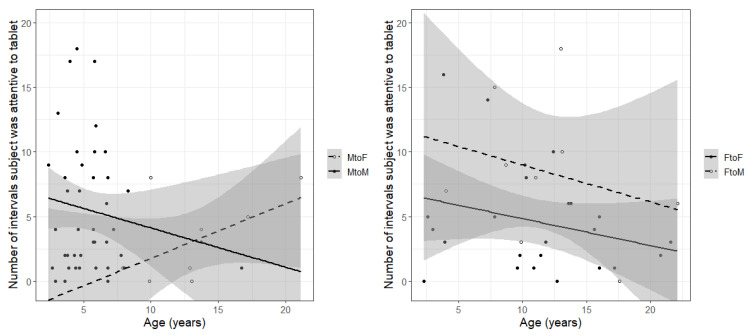
Number of intervals (out of 30) in which individuals were attentive to the iPad during Zoom session as a function of individual age and sex, with trendlines and 95% confidence intervals. (**Left**) panel shows males in Zoom sessions with female (dashed) or male (solid) partners. Young males were more attentive toward males than toward females, whereas older males were more attentive toward females than toward males. (**Right**) panel shows females in Zoom sessions with female (solid) or male (dashed) partners. Females were more attentive toward males than toward other females, and older females were less attentive overall than younger females.

In general, animals spent relatively little time attending to the iPad. All but eight animals (three females, five males) looked at it at least once during the 10 min session. The total amount of attention shown by individuals ranged from 0 to 18 intervals (i.e., 0–60% of intervals), with a median of 4 intervals (i.e., approximately 13% of the 30, 20 s intervals). The best model explaining the amount of attention had an AIC 14.5 points lower than the next best model and was significantly different (chi-square = 20.52, *p* < 0.001). Sex and age, as well as a sex-by-age interaction, explained attention shown (Table 1). However, the coefficient estimate of MtoF is negative (B = −3.26, *p* < 0.001), this is offset by the positive coefficient of the age by MtoF interaction (B = 0.22, *p* < 0.001), making the overall effect of MtoF positive. Thus, the highest levels of attention were displayed from male to female recipients, and this increased with male age (Figure 1). The amount of attention males showed to other males (B = −0.04, *p* > 0.05), and the amount that females showed to either male or female recipients (B = −0.11, *p* > 0.05; B(intercept) = 2.12, *p* < 0.001) was not significantly different from one another, and decreased with a main effect of age (B = −0.07, *p* < 0.01).

#### 3.1.2. Anxiety

**Figure 2:**
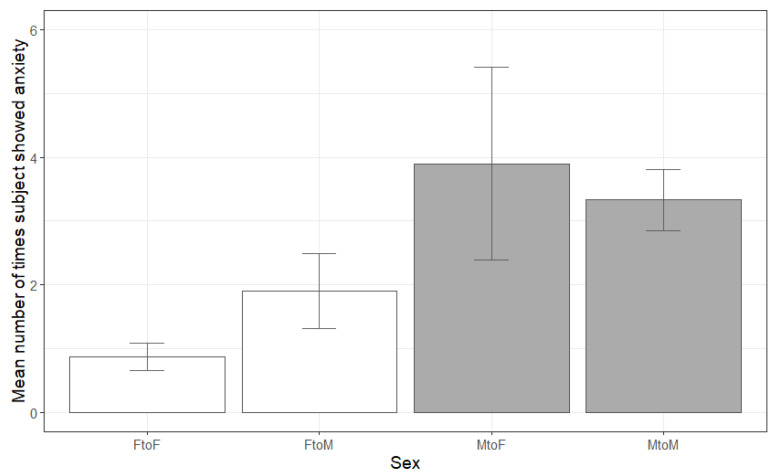
Mean and standard error of number of times individuals showed anxiety during the 10 min Zoom session based on sex of the individual and Zoom partner. On average, males showed more anxiety than females, regardless of whether the Zoom partner was male or female.

The total amount of anxiety shown by individuals ranged from 0–15 instances, with a median of 2 instances. Three models explained the amount of anxiety equally well when evaluated by AIC and chi-square tests. The model with the lowest AIC had a main effect on sex but no effect on age. The other two models (one with a main effect of age, the other with a main effect of age and an age by sex interaction) were within 2 AIC points of the sex-only model (1.2 and 1.5 points, respectively) and were not significantly better than the sex-only model (chi-square = 0.8, *p* > 0.1; chi-square = 6.5, *p* > 0.1). Since neither main nor interaction effects of age were significant in either model, we report only the sex-only model here (Table 2). Males showed more anxiety toward females (B = 1.43, *p* < 0.001) and males (B = 1.29, *p* < 0.001) than females showed to females. Tukey’s HSD contrasts additionally showed that males showed more anxiety to females than females showed to males (Tukey’s HSD = 0.72, *p* = 0.048). However, there was no difference in anxiety shown by males to either male or female recipients (Tukey’s HSD = −0.14, *p* > 0.1) or from females to either female or male recipients (Tukey’s HSD = −0.71, *p* > 0.1). Thus, males showed more anxiety than females overall, and anxiety did not depend on the sex of the Zoom partner (Figure 2).

#### 3.1.3. Prosocial

**Figure 3:**
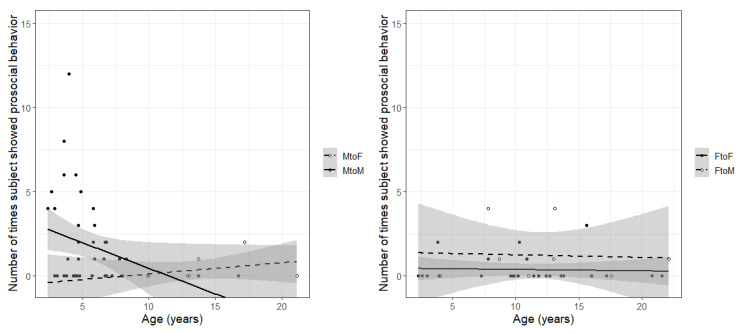
Number of times individuals showed prosocial behavior during 10 min Zoom session, as a function of individual age and sex of the individual and Zoom partner, with trendlines and 95% confidence intervals. (**Left**) panel shows males in Zoom sessions with female (dashed) or male (solid) partners. Young males in sessions with other males showed more prosocial behavior than older males in sessions with females. (**Right**) panel shows females in Zoom sessions with female (solid) or male (dashed) partners. Females tended to show more prosocial behavior toward a male partner than a female partner, regardless of female age.

The monkeys in this study showed little prosocial behavior during the Zoom sessions. The total amount of prosocial behavior shown by individuals ranged from 0 to 12 instances, with a median of 0 instances. Thirty-four subjects showed some prosocial behavior, but the remaining 50 subjects did not. The best model for prosocial behavior was 9.9 AIC points lower than the next best model (chi-square = 15.93, *p* < 0.01) and contained the main effects of age and sex, with an interaction between age and sex (Table 3). The most prosocial behavior was shown between pairs of males (B = 3.47, *p* < 0.01), but this decreased with male age (B = −0.45, *p* < 0.01) (Figure 3).

### 3.2. Relationship between Behavior on Zoom and Pair Outcome

Of the 42 pairs involved in this study, 28 (7FF, 6MF, and 15MM) were successfully socialized (i.e., co-housed for at least 14 days), while 14 pairs (5FF, 3MF, 6MM) showed immediate aggression when introduced and thus were not co-housed.

The initial comprehensive model was significantly different from models with fewer variables (chi-square = 10.5, *p* < 0.05) and had the lowest AIC by at least 4.5 points; thus, we report the initial comprehensive model. This model (Table 4) included significant effects of pair sex and attention similarity, with trends for total anxiety, sex by attention, and sex by anxiety interactions. The model predicted that, on average, MM pairs are more likely to be successful than FF pairs, which matches the pattern of successful pairs within our subjects.

Both in our sample and according to the model, MF pairs have a high likelihood of success. However, the model estimate for MF pairings had an outsized standard error (19.48) and was not statistically significant (*p* = 0.11). This may be a consequence of the lower number of MF pairs relative to other pair types and the number of successful pairs within that category.

The within-pair similarity of attention was the most impactful behavioral indicator of pair success. Predicted success increased when attention was dissimilar within the pair. FF pairs with similar attentiveness had a 0.4% (95% CI: 0.0–52.8%) likelihood of success, compared to a 30.4% (95% CI: 1.3–93.2%) likelihood of success when within-pair attentiveness was dissimilar. MM pairs with similar attentiveness had a 23.3% (95% CI: 3.4–72.3%) likelihood of success, whereas MM pairs with dissimilar attentiveness had a 97.0% (95% CI: 33.8–99.9%) likelihood of success. Thus, pairs in which attention was primarily shown by one animal had a higher chance of success than those in which both individuals showed similar levels of attention toward the iPad. There was a trend toward success for FF pairs that showed low anxiety (B = −1.95, *p* = 0.07), but for MM pairs that showed more anxiety (B = 2.09, *p* = 0.07), although these effects were not statistically significant. Neither prosocial behavior nor lag time were predictors of success in this sample.

## 4. Discussion

Social housing is widely accepted as an integral part of behavioral management with indoor-housed nonhuman primates [8]. However, finding appropriate partners when NHPs are housed in separate rooms or buildings adds unique challenges to compatibility assessments. Further, the introduction itself can be more stressful when partners are located apart from one another due to the relocation(s) involved for one or more animals [15]. Having the ability to assess compatibility prior to relocating animals for introductions may help address these concerns. Macaques show measurable interest in videos of conspecifics [18] to the point of finding them rewarding [20]. Therefore, we examined the use of live video conference technology (Zoom) as a tool for the initial introduction and compatibility assessment between potential partners in rhesus macaques.

Overall, the monkeys in our study spent relatively little time looking at the screen during the Zoom sessions. The maximum any animal spent looking at the iPad was 60% of intervals. Males with female partners spent more time looking at the screen than males with male partners or females with male or female partners. There was no difference in the amount of time females spent attending to the iPad screen based on the sex of their partner. Attention toward the screen generally decreased with age, with the exception of males who were in a session with a female; for those males, attention increased with age. However, this result should be interpreted with caution, as the sex of the partner we chose for male monkeys was, in part, based on his age. Males with female partners tended to be older than those with male partners (average age of males involved in MF pairs = 13 years +/− 4.1 years; in MM pairs = 6 years +/− 2.4 years). Thus, the increase in attention seen with age for males in MF pairs likely had to do with the sex of the partner and not the age of the male per se. These results are similar to those of a study examining social vigilance in semi-free-ranging rhesus macaques [29]. In that study, males showed more social vigilance than females, which the authors attributed to differences in social information gathering in the species. Further, those monkeys showed a decrease in social vigilance with age, and there was an effect of dominance rank, with high-ranking individuals more vigilant than lower-ranking monkeys [29]. The monkeys in our study were single-housed at the time of the Zoom session but had all been socially housed in the past. We do not know their previous dominance rankings or whether that would affect their current attention to social partners.

We focused on anxiety and prosocial behaviors for this study because those behaviors have been shown to predict pair success when seen early in the pair attempt. MacAllister and colleagues found that prosocial behaviors exhibited by the pair on the first day of the introduction were predictive of pair success, while pairs that exhibited anxiety behaviors were often unsuccessful [21]. Monkeys in our study showed relatively little anxiety or prosocial behavior, which is not surprising given the minimal amount of time they attended to the tablet. Males showed more anxiety than females overall, and anxiety did not depend on the sex of the Zoom partner. Interestingly, males with male partners showed more prosocial behavior, but less attention, than males with female partners. It is possible that this difference was related to the age of the monkeys; as stated above, males in male–male pairs were younger than those in male–female pairs. It could be that younger animals are more likely to show prosocial behavior.

Our focus for undertaking this study was to examine whether allowing animals to interact via video conference technology could help us predict pair compatibility. For example, if one or both animals threatened the other, that could indicate that they might not be compatible. We predicted that pairs that spent time paying attention to each other, particularly if they demonstrated prosocial behavior, would be more successful than those that did not. While the total amount of time animals spent looking at the iPad did not predict pair success, the within-pair similarity of attentiveness (e.g., amount displayed by the partners relative to one another) did. Pairs in which attention was primarily shown by one animal had a higher chance of success than those in which both individuals showed similar levels of attention toward the iPad for both MM and FF pairs. It is possible that this discrepancy is related to a clear dominance relationship for the potential partners, which has been found to help predict pair success in some studies (e.g., [3]).

Interestingly, prosocial behavior exhibited by the pair did not predict pair success. This finding may be due to the low amount of prosocial behavior displayed in general. Our Zoom sessions were relatively short and thus may not have been long enough to allow for the expression of prosocial behavior. Alternatively, it is also possible that the inability to follow prosocial behavior with physical contact diminished its effect on pair success.

We had also predicted that pairs that demonstrated aggressive or anxious behaviors would be less likely to be successful. We saw very little aggressive behavior; only three animals demonstrated any aggression at all during the Zoom sessions. There was a trend toward pair success in FF pairs that showed low anxiety (B = −1.95, *p* = 0.07) and MM pairs that showed increased anxiety (B = 2.09, *p* = 0.07), although these effects were not statistically significant. Anxiety behaviors could indicate either stress due to the inability to avoid the Zoom session entirely or frustration at an inability to have physical contact with the Zoom partner. Thus, anxiety could have actually indicated compatibility for some monkeys. Future studies should examine anxiety in conjunction with social vigilance to parse out these differences.

There were a number of potential confounds and limitations to this study that may have prevented us from finding more striking results. We only provided animals with one ten minute Zoom session due to logistical challenges and intermittent Wi-Fi availability. Longer and/or more frequent sessions most likely would have resulted in more robust findings. The iPad was novel for most individuals, and it is possible the animals could have been wary of the tripod set-up or unaware that they could interact with the tablet. A short training or introduction session to the equipment could potentially eliminate this particular confound. Similarly, the Zoom session was conducted on relatively small tablets. Larger iPads may have allowed the animals greater visual access. In addition, attention to the screen was scored from recordings. While the observer was highly trained, she was not in the room with the monkey during the Zoom session and only scored attention when it was clearly directed toward the screen. Thus, we may have underestimated the amount of time the animals spent looking at the iPad. Using eye-tracking technology may provide more precise data regarding social attention (e.g., [30,31]). Other confounds include the time of day when sessions were held. We did not keep to a strict window of time in which to perform Zoom sessions due to husbandry or project-related scheduling conflicts. Some sessions were performed close to normal feeding and enrichment times, which could introduce more opportunities for distraction.

Despite these confounds, our initial data suggest that video conferencing technology may be useful as a tool for introducing unfamiliar partners ahead of their pair attempt. There are also other uses for this technology. In many facilities, partners need to be temporarily separated from one another for research or clinical-related reasons. In these situations, Zoom sessions may be set up between the familiar animals so that they may communicate, thus minimizing the impact of physical separation. At the ONPRC, group-housed rhesus macaques who are injured or sick may be removed from their pen and brought to the clinical hospital. We have used Zoom to allow these monkeys to communicate with their groupmates while they are under clinical care. While we have only used this for a handful of animals so far, we have found that both the sick/injured monkey as well as the groupmates pay a great deal of attention to the screen. More work is needed to examine whether the ability to interact with groupmates affects how well an individual responds to medical treatment and the individual’s integration back into the group after medical treatment is completed.

## 5. Conclusions

In order to assess the compatibility of macaques prior to social introductions, the animals need to have the opportunity to interact with one another. At many facilities, that means moving animals from one room to another. These movements can be disruptive to the monkeys, and the stress can be compounded if the introduction is not successful. Having a way to assess the compatibility of potential partners prior to moving them could help to reduce unnecessary moves (i.e., if the partners are not compatible), therefore reducing stress. Video conference technology provides opportunities for nonhuman primates to interact with one another in real time. Studies have demonstrated that rhesus macaques show interest in video images of conspecifics (e.g., [16,17,18]); thus, it makes sense that they might also interact with conspecifics in this format. In our study, over 90% of the monkeys paid attention to the screen at some point during the Zoom session, and some even engaged in prosocial behavior toward the screen. We hypothesized that pairs in which the partners demonstrated affiliative behaviors during the Zoom session would be more likely to be successfully pair-housed than those in which there was no prosocial behavior exhibited. We did not find a correlation between prosocial behavior and pair success in our study, perhaps because they spent relatively little time engaged in affiliative behavior. However, pairs in which attention to the tablet was primarily shown by one animal had a higher chance of success than those in which both individuals showed similar levels. While more work needs to be done, our results are promising and suggest that this technology may be useful not only for initial compatibility assessments but also may help animals interact with conspecifics that are not located in visual contact.

## Figures and Tables

**Table 1:** Estimates from the Poisson regression for the number of intervals during which an individual was attentive to the iPad during the Zoom session.

	Estimate	Std. Error	*p*
Intercept	2.12	0.330	<0.001
Age	−0.07	0.025	0.004
Sex (FtoM)	0.01	0.584	ns
Sex (MtoF)	−3.31	0.846	<0.001
Sex (MtoM)	0.02	0.430	ns
Age × Sex (FtoM)	0.06	0.044	ns
Age × Sex (MtoF)	0.23	0.056	<0.001
Age × Sex (MtoM)	−0.04	0.051	ns

**Table 2:** Estimates from the Poisson regression for the number of times an individual showed anxiety during the 10 min Zoom session.

	Estimate	Std. Error	*p*
Intercept	−0.29	0.289	ns
Sex (FtoM)	0.68	0.431	ns
Sex (MtoF)	1.38	0.395	<0.001
Sex (MtoM)	1.30	0.328	<0.001

**Table 3:** Estimates from the Poisson regression for the number of times an individual showed prosocial behavior during the 10 min Zoom session.

	Estimate	Std. Error	*p*
Intercept	−1.07	1.005	ns
Age	−0.05	0.083	ns
Sex (FtoM)	1.55	1.605	ns
Sex (MtoF)	−5.03	3.470	ns
Sex (MtoM)	3.47	1.202	0.004
Age × Sex (FtoM)	−0.03	0.126	ns
Age × Sex (MtoF)	0.35	0.223	ns
Age × Sex (MtoM)	−0.45	0.154	0.003

**Table 4:** Estimates from the logistic regression of the success of pair introductions following the Zoom session.

	Estimate	Std. Error	*p*
Intercept	2.63	2.564	ns
Lag Time	−0.06	0.077	ns
Pair Sex (MF)	30.77	19.479	ns
Pair Sex (MM)	−8.58	4.142	0.038
Pair Anxiety	−1.95	1.084	0.072
Pair Attentiveness	−0.06	0.162	ns
Attentiveness Similarity (dissimilar)	4.66	2.155	0.031
Pair Prosocial	0.89	0.633	ns
Pair Sex (MF) × Pair Anxiety	0.07	1.368	ns
Pair Sex (MM) × Pair Anxiety	2.09	1.141	0.067
Pair Sex (MF) × Pair Attentiveness	−1.55	0.867	0.074
Pair Sex (MM) × Pair Attentiveness	0.24	0.254	ns

## Data Availability

The data presented in this study are available on request from the corresponding author.
